# Mechanisms of portal vein tumour thrombus formation and development in patients with hepatocellular carcinoma

**DOI:** 10.1111/jcmm.17808

**Published:** 2023-06-22

**Authors:** Zhenli Li, Mingda Zhao, Xingshun Qi, Yufu Tang, Shuqun Cheng

**Affiliations:** ^1^ Department of Hepatobiliary Surgery General Hospital of Northern Theater Command Shenyang China; ^2^ Department of General Surgery The 963rd Hospital of the Joint Service Support Force of the PLA Jiamusi China; ^3^ Dalian Medical University Dalian China; ^4^ Department of Gastroenterology General Hospital of Northern Theater Command Shenyang China; ^5^ Sixth Department of Liver Surgery, Eastern Hepatobiliary Surgery Hospital Second Military Medical University Shanghai China

**Keywords:** hepatocellular carcinoma (HCC), mechanism, portal vein tumour thrombus (PVTT)

## Abstract

Hepatocellular carcinoma (HCC) is one of the most common and aggressive human malignancies worldwide. Portal vein tumour thrombus (PVTT) is considered one of most fearful complications of HCC and is strongly associated with a poor prognosis. Clarification of the mechanisms underlying the formation and development of PVTT is crucial for developing novel therapeutic strategies for HCC patients. Several studies have been made to uncover that tumour microenvironment, stem cells, abnormal gene expression and non‐coding RNAs deregulation are associated with PVTT in patients with HCC in the last decade. However, the exact molecular mechanisms of PVTT in patients with HCC are still largely unknown. In the present review, we briefly summarized the molecular mechanisms underlying the formation and development of PVTT in HCC.

## 1 INTRODUCTION

Hepatocellular carcinoma (HCC) is one of the most common and fatal malignancies worldwide.^1^ Despite advances in the diagnostic and treatment strategies for different HCC stages, the five‐year survival remains extremely low.^2^ The presence of portal vein tumour thrombus (PVTT), which is not uncommon, has been identified as one of the most significant factors for poor prognosis in HCC. If left treatment, the median survival time for patients with PVTT is 2.7–4 months, compared with 10–24 months for patients without PVTT.^3^, ^4^ The great efforts have been performed to prolong the survival time of HCC patients with PVTT in the past several decades. Unfortunately, the survival benefit of the various treatment strategies is still extremely limited.^5^, ^6^, ^7^ Molecular‐targeted cancer therapy, which is ascribed to the increasing understanding of molecular mechanisms for cancer initiation and progression, is considered as a breakthrough for cancer therapy. However, sorafenib and lenvatinib, as molecular‐targeted agents recommended for the advanced HCC, are only able to slightly prolong overall survival compared to placebo in HCC patients with PVTT.^8^, ^9^, ^10^, ^11^, ^12^, ^13^ Therefore, clarification of the mechanisms underlying the formation and development of PVTT is crucial for developing novel therapeutic strategies for HCC patients.

**FIGURE 1:**
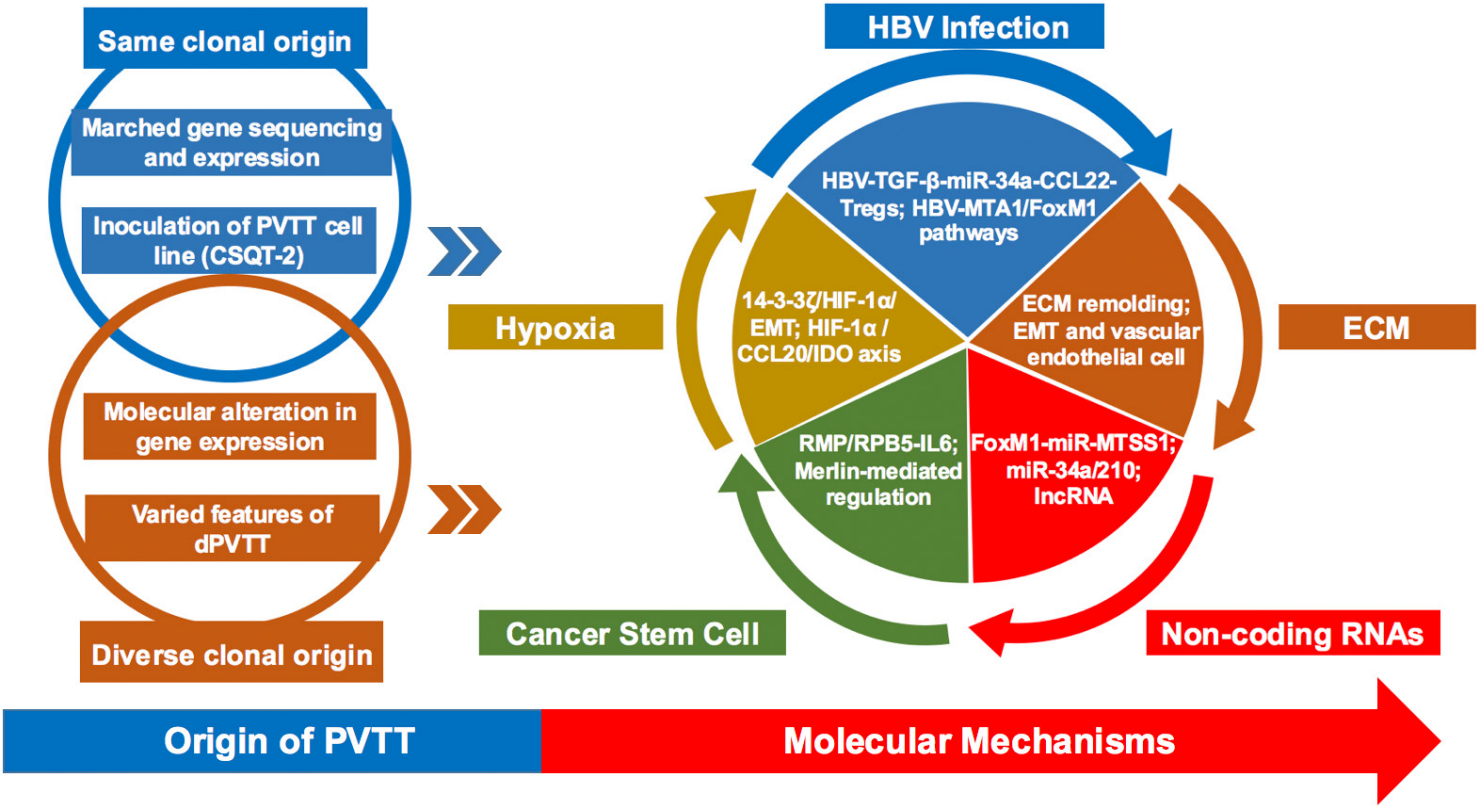
Schematic illustration of the molecular mechanism in the formation and development of PVTT. dPVTT, distinct PVTT; ECM, extracellular matrix; EMT, epithelial‐mesenchymal transition; HBV, hepatitis B virus; PVTT, portal vein tumour thrombus.

Multiple mechanisms underlying the formation and development of PVTT in HCC have been reported recently. It is well‐accepted that PVTT could originate from HCC primary nodules by metastasis, and evidence from several studies showed that gene expression profiles of PVTT tissues (PVTTs) were nearly identical to the corresponding primary HCC tissues (HCCs).^14^, ^15^, ^16^, ^17^, ^18^ However, previous studies also showed different gene profiles between PVTTs and HCCs, suggesting that PVTTs do not always have clonal origin from their paired HCCs. Furthermore, tumour microenvironment (including hepatitis B virus (HBV)‐associated microenvironment, hypoxia, and extracellular matrix (ECM)), cancer stem cells and non‐coding RNAs have been found to contribute to PVTT development.^19^, ^20^, ^21^, ^22^, ^23^, ^24^, ^25^ It is noteworthy that the mechanism of PVTT formation and development is far more complicated than previously thought and further great efforts need to be performed. Thus, in this review, we summarize the current state of knowledge and highlight the mechanisms underlying the formation and development of PVTT (Figure 1 and Table 1).

**TABLE 1:** Mechanism in the formation and development of PVTT in patients with HCC.

	Evidence		Evidence
HBV and PVTT		ECM and PVTT	
Features	Impact of HBV infection and AVT on PVTT^40^, ^41^, ^42^, ^43^, ^44^, ^45^	Features	ECM‐mediated transformation in tumour microenvironment and PVTT^16^, ^25^, ^73^, ^74^
Molecular mechanisms	HBV‐TGF‐β‐miR‐34a‐CCL22‐Tregs^21^ HBV‐MTA1 /FoxM1 pathways^46^, ^47^, ^48^	Molecular mechanisms	ECM remoulding,^16^, ^75^, ^76^ EMT,^78^ and transformed vascular endothelial cell^83^
Hypoxia and PVTT		CTCs and PVTT	
Features	Intense correlation of HIF‐1α signalling pathways and PVTT^59^, ^60^	Features	Stem cell and EMT traits^90^
Molecular mechanisms	Hypoxia/14–3‐3ζ/HIF‐1α/EMT pathway,^19^ Hypoxia/HIF‐1α /CCL20/IDO axis^20^	Molecular mechanisms	Interaction between portal vein endothelial cells and CTCs^91^, ^92^, ^93^
CSCs and PVTT		Non‐coding RNAs and PVTT	
Features	Biomarkers included CD133, CD90, EpCAM, CD44 and CD24^22^, ^67^, ^68^	Features	Varied expression levels of non‐coding RNAs in venous metastases tissue^17^
Molecular mechanisms	RMP‐P65/RPB5‐IL6 interaction; ICR‐ICAM‐1^22^; ^wt^Merlin and ^Δ2–4^Merlin‐mediated regulation^70^	Molecular mechanisms	FoxM1‐miRNA‐135a‐MTSS1^24^; HBV‐TGF‐β‐miR‐34a‐CCL22‐Tregs^21^

Abbreviations: AVT, antiviral treatment; CSC, cancer stem cells; CTC, circulating tumour cell; ECM, extracellular matrix; EMT, epithelial‐mesenchymal transition; HBV, hepatitis B virus; HIF‐1α, hypoxia‐inducible factor alpha; IDO, indoleamine 2,3‐dioxygenase; PVTT, portal vein tumour thrombus.

## 2 ORIGINS OF PVTT

PVTT commonly occurs in patients with HCC. Approximately 10%–40% of HCC is accompanied by PVTT when they are first diagnosed,^26^ while up to 44.0%–62.2% at autopsy.^27^ Considering such a relatively high prevalence of PVTT among HCC patients, it has been usually considered as a special type of HCC intrahepatic metastasis in the past several decades. Evidence from several studies also identified PVTT originating from HCC primary nodules by metastasis. Direct evidence from our previous study showed that a PVTT‐originated HCC cell line (CSQT‐2), established by our group, was able to generate PVTT in nude mice after orthotropic inoculation, indicating that PVTT could be formed by HCC cells disseminating from the primary tumour site.^28^ Genomic analysis of the paired tumour tissues from HCC patients would uncover whether PVTT originate from the primary HCC tissues. Ye et al.^14^ compared the gene expression profiles of 67 primary and metastatic (including PVTTs) HCCs samples from 40 patients using cDNA microarray technology, and uncovered that the gene expression signature of primary HCCs was very similar to that of their paired metastatic HCC samples. Lin et al.^29^ also found that there were not many differentially expressed genes between HCC and paired PVTT tissues. Huang et al. compared the genetic alterations of HCCs from 10 patients with that of paired PVTTs, and then validated by targeted sequencing in a larger set of 100 paired samples. They found that somatic mutations in 347 genes were identified in the HCCs and/or PVTTs, and more than 94% somatic mutations were shared by HCCs and paired PVTTs. Wong et al. analysed the MicroRNA (miRNA) expressions profiles among non‐tumorous livers, primary HCCs and PVTTs in the same livers from 20 HCC patients by TaqMan low‐density array. They found that the miRNA expression profiles of PVTTs were nearly identical to their corresponding primary HCCs.^18^ Moreover, the transcriptomic signatures of HCC with PVTT patients have been explored using RNA sequencing. The results from this study also identified the PVTTs transcriptomic were similar to their paired HCCs.^16^ All of these studies supported a viewpoint that PVTT derived from malignant cells within the primary tumours. Nevertheless, different evidence from recent studies indicated that PVTTs may have a different clonal origin than that of the corresponding HCCs. Some researchers found few differentially expressed genes between PVTTs and their corresponding HCCs. Wang et al. collected matched adjacent normal, primary tumour and PVTT samples from 19 HCC patients, and investigated the molecular alterations in these matched samples. They used an individualized expression analysis to identify the differentially expressed genes between PVTTs and corresponding HCCs for each patient. The results showed that 8 of out 19 patients had nearly identical gene expression between PVTTs and corresponding HCCs, and five patients acquired evidential progressive alterations of gene expressions (more than 1000 differentially expressed genes were identified in each patient).^15^ Based on the results, Wang et al. speculated that most patients had few differentially expressed genes, indicating that PVTTs may be formed by the accumulation of randomly fallen cancer cells from primary HCC tissues. They also considered that PVTTs might be formed by highly invasive sub‐clones or the randomly fallen cancer cells acquired adaptive changes for the portal vein microenvironment in those patients with evidential progressive alterations of gene expressions. Indeed, tumour microenvironments, especially hypoxia microenvironment, played a crucial role in regulating genes expression in tumour patients.^30^ However, the number of evidential progressive alterations was larger than that of tumour microenvironment‐related genes reported previously.^31^, ^32^ Hence, this possibility that PVTTs may have different clonal origins from their corresponding HCCs in those patients with evidential progressive alterations cannot be ruled out. Moreover, in a previous study from our group, the clonal relationship of PVTTs with corresponding HCCs was analysed using datasets deposited in a public database. We found that one out of 19 PVTTs was identified to have an independent clonal origin from its corresponding HCCs, and the PVTTs with independent clonal origin showed different gene expression and enrichment in biological processes from the primary HCCs.^33^ Furthermore, a distinct PVTT (dPVTT), distant from or without liver parenchyma tumour nodules, has been observed in some patients.^34^, ^35^, ^36^ To identify the biological features of dPVTT in HCC patients, we collected the matched tumour tissues from 5 HCC patients accompanied by dPVTT in our library, and two‐dimensional electrophoresis has been performed to compare the proteome of dPVTT tissues with that of corresponding HCCs. The differentially expressed proteins were identified in dPVTT tissues and HCCs, and protein expression levels with differences of more than threefold were found in at least 80% of the patients. Importantly, we demonstrated that dPVTT showed a more malignant phenotype by detecting the expression of C‐Kit.^34^ This finding further indicated that some PVTTs did not have the same origin as the paired tumours. On a more serious note, several studies also identified PVTTs from different HCC patients showed very different gene expression profiles,^15^, ^17^, ^33^ implying that the mechanism of PVTT formation is far more complicated than previously thought, and great efforts need to be performed to clarify these inferences further.

## 3 HBV INFECTION AND PVTT

A mountain of evidence has shown that HBV infection was strongly associated with HCC formation and development, and more than 50% of HCC cases may be caused by persistent HBV infection.^37^, ^38^ The potential correlation between HBV infection and the formation and development of PVTT also has been investigated for HCC patients in the last decades. In a nationwide study involving 11,950 patients with HCC, the clinicopathological features were compared among HBV‐related HCC, hepatitis C virus (HCV)‐related HCC and Non‐B Non‐C HCC patients. The results showed that the incidence of PVTT in HBV‐related HCC patients (31%) was higher than that in HCV‐related HCC patients (22%) and Non‐B Non‐C HCC patients (29%).^39^ The similar results from other clinical studies also uncovered that those patients with HBV‐related HCC were more likely to develop PVTT or microvascular invasion (MVI, also named as microscopic PVTT) than patients with HCC with other aetiologies. The evidence from several other clinical studies uncovered that the status of HBV infection was associated with the development of vascular invasion (including PVTT and MVI), and preoperative antiviral treatment (AVT) would decrease the risk of vascular invasion.^40^, ^41^, ^42^ In a MVI risk estimation nomogram, a high seral HBV DNA load (>10^4^ IU/mL) has been reported as an independent factor predicting the presence of MVI(OR = 2.33). To identify whether preoperative AVT can affect the incidence of MVI in HBV‐related HCC, a larger cohort of HCC patients (*n* = 2362) was analysed by Li et al., revealing that a higher preoperative HBV DNA level (≥2000 IU/mL) was associated with an increased risk of MVI (OR = 1.399) in those non‐AVT patients. Importantly, a lower incidence of MVI has been found in AVT patients than that in non‐AVT patients (38.7% vs. 48.6%, *p* = 0.001), and AVT has been demonstrated as a protective factor of MVI (OR = 0.758) and early tumour recurrence (OR = 0.732). The other two studies further showed the similar conclusions that HBV replication was an independent risk of vascular invasion and AVT had an inhibitory effect on vascular invasion formation.^43^, ^44^ In addition, in a retrospective study that included 486 patients, we reported that the serum HBV levels might be associated with the extent of vascular invasion, and AVT could suppress the development of vascular invasion in patients with HBV‐related HCC.^45^

Although those clinical studies identified the association of HBV infection and PVTT formation, the molecular mechanisms are still extremely unclear. Therefore, to explore the molecular mechanisms underlying the association between HBV infection and PVTT formation in HCC patients, we participated into a study conducted by researchers at Duke University.^21^ Among 288 HCC patients, we found a strong correlation between HBV status and the presence of PVTT, concomitant with elevated TGF‐β activity. When screening microRNAs related to metastasis, we found that a reduced level of miR‐34a, a tumour suppressor, was directly linked to high TGF‐β activity. Moreover, the chemokine gene CCL22 has been identified as a primary target miR‐34a in PVTT‐originated HCC cells. Using in vitro assays and mouse models of liver or lung metastasis, we demonstrated that TGF‐β signalling, via miR‐34a suppression and consequent elevation of CCL22, enhanced recruitment of immune suppressive CD4^+^CD25^+^ T regulatory (Tregs) cells to create an immune suppressive microenvironment, thereby promoting metastasis. These findings revealed a novel and important molecular mechanism underlying PVTT development by HBV‐TGF‐β‐miR‐34a‐CCL22‐Tregs pathway. Moreover, HBV X protein (HBX), a key regulatory multifunctional protein of the virus, may contribute to the vascular invasion of HCC. Metastatic tumour antigen 1 (MTA1), upregulated by HBX,^46^, ^47^, ^48^ has been reported to be strongly correlated with MVI and early recurrence in patients with HCC.^49^ HBX also involved in elevated expression of Forkhead box M1 (FoxM1),^50^ which has been identified to play an important role in promoting the development of vascular invasion.^24^, ^50^ Although these findings, to a certain extent, explained how HBV infection promoted the formation and development of PVTT in patients with HCC, we believe that some issues remain unclear or unanswered and we are seeing only the tip of iceberg.

## 4 HYPOXIA AND PVTT

Hypoxia, one of the hallmarks of tumours, is common in tumours including HCC. Hypoxia can potentially regulate every aspect of cellular function including growth, proliferation, apoptosis, metastasis, immunity, metabolic reprogramming, self‐renewal and others.^51^, ^52^ Hypoxia is associated with resistance to chemotherapy and radiotherapy, and is closely related to poor prognosis of HCC. In HCC, hypoxia results from a shortage of blood circulation caused by liver cirrhosis and the rapid growth of tumour cells. Unsurprisingly, liver cirrhosis and tumour size (>8 cm) are both independent predictors of PVTT in HCC.^53^ In addition, expression of protein disulfide‐isomeraseA6 (PDIA6), apolipoprotein A‐I (APO A‐I) and CXC chemokine receptor 4 (CXCR4) has been identified to correlate with the presence of PVTT,^54^, ^55^ and can also be induced by hypoxia.^56^, ^57^, ^58^ Importantly, elevated hypoxia‐inducible factor alpha (HIF‐1α), an important transcription factor involved in the hypoxic response of cells, has been found to closely relate to PVTT and poor prognosis in HCC patients.^59^, ^60^ Based on these findings, the association between hypoxia and PVTT formation aroused our interests, and some related researches have been performed in our laboratory. Among 143 patients with HCC, we uncovered a causative link between intratumoral hypoxia and PVTT formation. In an analysis of HIF‐1α targeted proteins related to HCC progression, we found elevated levels of 14–3‐3ζ were induced by hypoxia and correlated with PVTT formation in HCC patients. We then found 14–3‐3ζ upregulated HIF‐1α expression by recruiting HDAC4, which prevented HIF‐1α acetylation, thereby stabilizing the protein. This pro‐survival role of 14–3‐3ζ in stabilizing HIF‐1α was necessary for hypoxia‐induced expression of genes, including those indicative of epithelial‐mesenchymal transition (EMT), which led to tumour metastasis.^19^ Our results established the hypoxia/14–3‐3ζ/HIF‐1α /EMT pathway, and we believe it plays an important role in HCC metastasis and PVTT formation. Furthermore, in the development of PVTT, a link between hypoxia and immunosuppressive tumour microenvironment has been identified in a previous study.^20^ This study demonstrated that the hypoxia‐induced EMT of cancer cells helped to educate newly recruited monocytes by secreting CCL22, which led to indoleamine 2,3‐dioxygenase (IDO) upregulation in the monocytes, and IDO^+^ monocyte‐derived macrophages exerted an immunosuppressive effect on T cells, which thus created a tumour immunosuppressive microenvironment and promoted tumour metastasis. Among 90 patients with HCC, high HIF‐1α expression strongly correlated with the presence of PVTT and poor prognosis, concomitant with elevated CCL22 expression, indicating that hypoxia/HIF‐1α/CCL20/IDO axis in HCC was important for promoting metastasis through inducing EMT and establishing an immunosuppressive microenvironment. Invasion of ECM is an early and essential step of the metastatic cascade. Hypoxia triggered invadopodia‐mediated ECM degradation may play an important role in HCC metastasis. Experiments in HCC cells by Kai et al. uncovered that TIMP2, a tissue inhibitor of metalloproteinases, was suppressed through a regulatory feedback circuit consisting of HIF‐1α/miRNA‐210/HIF‐3α under hypoxic environment. Importantly, the decreased incidence of venous invasion was observed in a *vivo* orthotopic tumour cell injection model, which is injected into HCC cells with ectopic expression of TIMP2.^61^ These findings further support the standpoint that hypoxia plays a crucial role in the progression of HCC, including PVTT formation or development.

Up to date, newly developed agents targeted at hypoxia‐signalling pathways have been attempted in clinical trials for HCC,^62^ which could be further used for PVTT patients. Since targeting HIFs or their signalling pathways would disrupt the cancer hallmarks, the novel inhibitors have shown promising effects in pre‐clinical and clinical trials for HCC management.^63^ RO7070179 is an antisense oligonucleotide that targets HIF‐1α in a synthetic locked nucleic acid form. Pre‐clinical studies showed a remarkable decrease in HIF‐1α mRNA level in HCC patients taking the RO7070179.^64^ Besides, a recent phase‐1b study demonstrated that HCC patients could get favourable response treated with RO7070179, indicating its great potential in HCC therapy.^64^ Another notable signalling pathway inhibitor of hypoxia is CT‐707, a potent Yes‐associated protein (YAP) signalling inhibitor.^65^ A phase‐1 clinical trial confirmed CT‐707 disturbed the IGF1R‐YAP axis under hypoxia HCC microenvironment, which might be applied as an effective therapeutic alternative for HCC with PVTT.^65^

## 5 CANCER STEM CELLS (CSCs) AND PVTT

CSCs, a subpopulation of cancer cells within a tumour, have typical characteristics related to stem cells. A growing body of evidence showed that CSCs were closely related to initiation, metastasis, relapse and chemoresistance in HCC, because of their capacity to self‐renew and pluripotency.^66^ Various surface molecules, such as CD133, CD90, epithelial cell adhesion molecule (EpCAM), CD44 and CD24, have been identified as candidate markers of CSCs for HCC. Previously, higher expression of EpCAM and CD24 has been found in the clinical PVTT tissues.^22^ In HCC, identification of EpCAM was associated with higher frequency of PVTT and significantly shorter survival.^67^ In a meta‐analysis, Zhong et al. found that CD133 overexpression was significantly associated with vascular invasion (OR = 2.06) and vascular thrombosis (OR = 1.47).^68^ CD90 also has been found to positively correlated with clinicopathologic characteristic, including pathological grade, satellite lesions and PVTT. Furthermore, we found almost the whole population of CSQT‐2 come from PVTT in cell line was EpCAM positive.^28^ All of these results indicated that CSCs may be involved in PVTT formation in HCC. A few researchers also attempted to explore the mechanisms underlying the relationship between CSCs and PVTT. As known, a study by Zhang et al. firstly uncovered a causative link between CSCs and PVTT formation. Mechanistically, RMP could interact with P65 and RPB5 in HCC cells, enhanced production of IL‐6, which resulted in maintaining of stemness and metastasis capacity, and thus promoting PVTT formation.^22^ In our study, intercellular adhesion molecule 1 (ICAM‐1) has been identified as a novel marker for HCC stem cells.^69^ Importantly, we further demonstrated ICAM‐1 involving in PVTT formation in HCC patients in another study. In a screen of paired long non‐coding RNAs (lncRNAs)‐mRNAs related to PVTT, we found that elevated levels of ICAM‐1 were regulated by ICAM‐1‐related non‐coding RNA (ICR). We also uncovered that ICR modulated the CSC properties of HCC cells by regulating ICAM‐1 expression, and ICR was regulated by Nanog, which played a pivotal role in CSC maintenance. In addition, both of ICAM‐1 and ICR were significantly elevated in PVTT tissues and were associated with poor prognosis in patients with PVTT.^23^ We previously reported that a splicing variant of moesin‐ezrin‐radixin‐like protein (Merlin), a tumour suppressor protein, promoted HCC metastasis and PVTT formation by interfering with the tumour suppression role of ^wt^Merlin. A novel splicing variant of Merlin, lacking the sequences encoded by exons 2, 3 and 4, has been designated as ^Δ2–4^Merlin, and closely correlated with PVTT formation in HCC patients. We found that ^wt^Merlin could bind to β‐catenin and ERM, but ^Δ2–4^Merlin lost this binding capacity, and ^Δ2–4^Merlin induced EMT by upregulating Twist expression. ^Δ2–4^Merlin elevation could increase self‐renew shown by more and bigger spheroids, potentially by upregulating the expression of β‐catenin and the nuclear accumulation and activities of other stemness genes (Sox2, OCT4, Klf4, C‐myc and Nanog).^70^

At present, numerous clinical studies of CSC‐based therapy for HCC have been explored and achieved encouraging results.^71^ The CSC‐based therapy consists of agents targeting CSC surface markers, targeting CSC‐intrinsic/−extrinsic regulators, as well as CSC‐directed immunotherapy.^71^ Among them, several molecular agents are involved in the targets of PVTT formation and development. For instance, ANXA3‐neutralizing monoclonal antibodies eradicated liver CSC subsets that expressed CD24, CD133 and EpCAM.^72^ As such, they could inhibit the growth and self‐renewal of HCC and be sensitive to the treatment of cisplatin and sorafenib, which exhibited great potential in the prevention and treatment of PVTT.

## 6 ECM‐MEDIATED TRANSFORMATION IN MICROENVIRONMENT AND PVTT

From the molecular and histopathological perspectives, ECM‐mediated transformation in tumour microenvironment is of paramount in the formation and development of PVTT.^16^, ^25^, ^73^, ^74^ This process is initiated by the remoulding of ECM with ECM‐receptor interaction.^16^, ^75^, ^76^ For protein digestion and absorption, cancer‐associated fibroblasts (CAFs) and macrophage type 2 cells in the ECM released large amounts of matrix metalloproteinases (MMPs). Then, MMPs transformed the ECM‐receptor interaction and stiffness of the ECM, which was associated with an increased risk of metastasis.^74^ Subsequently, increased matrix stiffness reinforced the polarization of M2 macrophages and their secretion of lysyl oxidase‐like 2 (LOXL2), further producing increased fibronectin and facilitating the metastasis.^77^ As such, the degradation and remoulding of ECM participated in the dynamic formation of PVTT.

Besides the remoulding of ECM, the EMT and transformation of vascular endothelial cell also play a critical role in the whole metastatic process from primary foci to portal vein.^78^ It has been confirmed that EMT participated in tumour progression by enhancing motility and invasive activity. Currently, there is no direct histopathological evidence to confirm the relationship between EMT and PVTT formation. However, at the level of molecular mechanism, the high expression of Cycle‐G1 in PVTT and the promotion of Cycle‐G1 in EMT by PI3K/Akt/GSK‐3/Snail pathway might suggest their potential correlation.^79^

In the transformation of vascular endothelial cell, latest basic researches observed high expression of receptor tyrosine kinase (AXL) in tumour‐derived endothelial cells (TECs), which was positively correlated with CD‐31 expression in vitro and in vivo.^80^ By activating the PI3K/Akt/SOX2/DKK‐1 axis, AXL stimulated proliferation and migration of TECs. Therefore, overexpression of AXL in TECs accelerated vascular metastasis of HCC.^81^ Additionally, vascular endothelial cells also involved in the EMT during HCC progression.^82^ In the process of classical EMT, N‐cadherin as a mesenchymal marker were increased, whereas E‐cadherin (one of epithelial markers) were reduced. With enhanced motility and invasive activity, these variations by vascular endothelial cells were considered to contribute to the formation and development of PVTT.^83^

## 7 CIRCULATING TUMOUR CELLS AND PVTT

CTCs are cancer cells in circulation dissociated from primary tumours. CTCs can be used as a biomarker to noninvasively monitor cancer progression and guide therapy.^84^ There is increasing evidence revealing that CTCs are closely related to recurrence and shorter recurrence‐free survival of HCC.^85^, ^86^, ^87^, ^88^, ^89^ Previous studies demonstrated that CTCs can be detected in blood sample from a considerable number of patients with early‐stage HCC or even in a small fraction of patients with HBV infection before small HCC detected by CT/MRI. These results suggested that tumour dissemination may be an early event in HCC pathogenesis. Moreover, CTCs are considered to be an active source of metastases due to their potential stem cell features and EMT traits, which allow them to disseminate effectively. From the perspective of tumour microenvironment, the generation of CTC is a part of the transformation of ECM and EMT.^90^ Researchers have noticed that CTCs obtained mesenchymal features via EMT, and then infiltrated the ECM by releasing proteolytic degradation enzymes and crossing the basement membrane. Eventually, they passed into the circulation and extravasated to form secondary micro‐metastasis.^91^, ^92^ However, whether PVTT originates from CTCs, and upon their entry into the bloodstream, how CTCs survive in portal vein and thereby formulate PVTT remain largely unknown. Based on the latest theory and researches, we speculate that the crosstalk between portal vein endothelial cells and CTCs may contribute to PVTT formation. In a recent study, the conditioned medium of vascular endothelial cells was used to culture HCC cells, finding that vascular endothelial cells can affect the cell adherence ability and viability of HCC cells, which was important for PVTT formation.^93^ The great efforts are needed to be focused on the molecular mechanisms of CTCs‐induced PVTT, which will provide more knowledge about PVTT formation.

Given the significant correlation between CTC and EMT, the CTC markers have been assessed in clinical studies as effective biomarkers of HCC.^94^ A recent study highlighted that mesenchymal‐CTCs gained mesenchymal features via EMT and promoted HCC metastasis.^94^ They could not only represented the progression and state of HCC, but also serve as a prognostic marker for long‐term survivals.

## 8 NON‐CODING RNAS AND PVTT

Non‐coding RNAs including miRNAs and lncRNAs are pivotal participants and regulators in the development and progression of HCC.^95^ The role of miRNAs and lncRNAs in PVTT formation also has been investigated in the last decade.^17^, ^18^, ^21^, ^24^, ^88^ One study uncovered a marked global reduction of miRNA expression levels in venous metastases tissue (including PVTT and hepatic vein tumoral thrombus), as compared with primary HCC tissue, and those global miRNAs have been found to have profound functions in regulating HCC metastasis. In a previous research conducted by our group, we found that miRNA‐135a was overexpressed in PVTT tissues and associated with the prognosis and survival of HCC patients with PVTT. Importantly, we demonstrated a novel regulatory pathway (FoxM1‐miRNA‐135a‐MTSS1) that could contribute to PVTT formation.^24^ Especially, as mentioned in the part of HBV‐related mechanism of PVTT, miRNA‐34a could regulate the HBV‐initiated formation of PVTT via mediating modified microenvironment by HBV‐TGF‐β‐miR‐34a‐CCL22‐Tregs pathway.^21^ MiRNA‐210 also has been identified to contribute to PVTT formation. Moreover, in a previous study, the expression profiles of lncRNAs have been investigated in HCC tissues and their paired PVTT tissues.^17^ Although the overall lncRNA expression patterns of PVTT were indistinguishable from those of their matched HCC tissues, approximately 100 lncRNAs were significantly deregulated in PVTT tissues in comparison with their expression levels in paired HCC tissues. In addition, those deregulated lncRNAs have been found to be correlated with cell adhesion, immune response and metabolic process. Ten lncRNA candidates have been selected in those deregulated lncRNAs and function assays have been performed, the results showed the candidate lncRNAs played essential roles in metastasis, indicating the deregulated lncRNAs have roles in PVTT formation.^17^ We found an ICR can regulate CSC properties of HCC cells and ICR contributes to PVTT development.^23^ Thus, we believe non‐coding RNAs play an important role in PVTT formation and development. Up to now, the regulatory effect of non‐coding RNA in the progression of HCC and PVTT has still been under experimental study, further investigations are required to pave the way for clinical translation.

## 9 CONCLUSIONS

Clearly, PVTT is common and it worsens prognosis of HCC. Although a mountain of evidence uncovered PVTT originated from the primary HCC tissues, several studies also identified that PVTT may have different clonal origins from their corresponding HCC. Molecular mechanisms in the formation and development of PVTT constituted a complicated regulatory network, including HBV infection, hypoxia microenvironment, CSCs, ECM, CTCs and deregulated non‐coding RNAs. However, the knowledge of the mechanisms underlying PVTT formation and development is only the tip of the iceberg, and it need to be further investigated and mined for optimizing therapeutic options for HCC, which may offer new approaches to improve the prognosis of HCC.

## AUTHOR CONTRIBUTIONS

**Zhenli Li:** Investigation (equal); methodology (equal); writing – original draft (equal). **Mingda Zhao:** Methodology (equal); writing – original draft (equal). **Xingshun Qi:** Writing – review and editing (equal). **Yufu Tang:** Conceptualization (equal); project administration (equal); writing – review and editing (equal). **Shuqun Cheng:** Conceptualization (equal); project administration (equal).

## FUNDING INFORMATION

This study was supported by the Natural Science Foundation of Liaoning Province (No. 2021JH2/10300117) and Postdoctoral Science Foundation of China (No. 2018T111168).

## CONFLICT OF INTEREST STATEMENT

The authors confirm that there are no conflicts of interest.

## Data Availability

Data openly available in a public repository that issues datasets with DOIs.
